# Modeling Associations between Principals’ Reported Indoor Environmental Quality and Students’ Self-Reported Respiratory Health Outcomes Using GLMM and ZIP Models

**DOI:** 10.3390/ijerph13040385

**Published:** 2016-03-30

**Authors:** Oluyemi Toyinbo, Markus Matilainen, Mari Turunen, Tuula Putus, Richard Shaughnessy, Ulla Haverinen-Shaughnessy

**Affiliations:** 1 National Institute for Health and Welfare, Kuopio FI-70701, Finland; manmat@utu.fi (M.M.); mari.turunen@thl.fi (M.T.); ulla.haverinen-shaughnessy@thl.fi (U.H.-S.); 2 Faculty of Medicine, Occupational Health Care Unit, University of Turku, Turku 20014, Finland; tuula.putus@utu.fi; 3 Indoor Air Program, the University of Tulsa, Tulsa, OK 74104, USA; rjstulsau@aol.com

**Keywords:** IEQ, schools, questionnaire, health, symptoms

## Abstract

*Background*: The aim of this paper was to examine associations between school building characteristics, indoor environmental quality (IEQ), and health responses using questionnaire data from both school principals and students. *Methods*: From 334 randomly sampled schools, 4248 sixth grade students from 297 schools participated in a questionnaire. From these schools, 134 principals returned questionnaires concerning 51 IEQ related questions of their school. Generalized linear mixed models (GLMM) were used to study the associations between IEQ indicators and existence of self-reported upper respiratory symptoms, while hierarchical Zero Inflated Poisson (ZIP)—models were used to model the number of symptoms. *Results*: Significant associations were established between existence of upper respiratory symptoms and unsatisfactory classroom temperature during the heating season (ORs 1.45 for too hot and cold, and 1.27 for too cold as compared to satisfactory temperature) and dampness or moisture damage during the year 2006–2007 (OR: 1.80 as compared to no moisture damage), respectively. The number of upper respiratory symptoms was significantly associated with inadequate ventilation and dampness or moisture damage. A higher number of missed school days due to respiratory infections were reported in schools with inadequate ventilation (RR: 1.16). *Conclusions*: The school level IEQ indicator variables described in this paper could explain a relatively large part of the school level variation observed in the self-reported upper respiratory symptoms and missed school days due to respiratory infections among students.

## 1. Introduction

In Finland, pupils spend an average of six hours a day on lessons in school [1], where they are exposed to their school environment. According to Sundell *et al.* [2], there is a need for thorough research on indoor environmental quality (IEQ) in schools because children are more susceptible to indoor pollution than adults. This is related to their immature tissues and organs which are still growing, they breathe in proportionately more air [3,4], and they have minimal control over their environment [4]. Thus far, a few studies have been done on school IEQ when compared to that of other buildings [5], and IEQ problems tend to persist as a result of low funding for research as well as maintenance of school buildings and facilities [6,7].

IEQ refers to the quality of a building’s environment in relation to the health and wellbeing of those who occupy space within it. IEQ is determined by many factors such as air quality and thermal conditions, which can be influenced by the condition and operation of heating, ventilation and air conditioning systems as well as occurrence of dampness or mold.

The composition of the outdoor environment may have an effect on IEQ as in the case of particulate matter from natural or artificial sources, while some IEQ parameters, such as certain volatile organic compounds (VOCs), may be exclusive of the outdoor environment [8]. Also, chemical processes between compounds present indoors can cause changes in IEQ [9].

The impact of building characteristics on IEQ cannot be overemphasized: the year of construction, state of repair, heating, ventilation and air conditioning (HVAC) systems and their maintenance, as well as materials used for the interior of building may affect the indoor environment. School buildings that are old may be contaminated with materials such as asbestos [10]. According to Espejord [11], inadequate insulation as well as leaky structures may increase heat loss. Kim and colleagues reported a significant amount of plasticizer and microbial volatile organic compounds (MVOCs) in newly constructed buildings due to emission from the materials used in the building construction [12].

ASHRAE standard 55 describes thermal comfort as a product of heat exchange of someone’s body with the immediate environment [13]. de Dear [14] concluded that it affects the overall performance of an individual. For thermal comfort to be acceptable for 85% of building occupants, it has been recommended that indoor temperature should always be 23 °C or lower [15] but not lower than 18 °C [16].

The type of ventilation system has been related to ventilation rate (L/s per student) in school buildings [17,18,19]. Classrooms in naturally ventilated schools were found to have increased levels of outdoor pollutants from traffic leading to asthma exacerbation in students [20]. Adequate ventilation can improve student learning, but the condition of the HVAC systems is also important [2]. A poorly maintained ventilation system may be a source of odorous and stuffy air [21]. Daisey *et al.* [22] found inadequate ventilation in some schools leading to health issues, and a recent study [23] associated inadequate ventilation with increased student absenteeism. Some measures that could reduce ventilation rates include energy conservation and HVAC system performing below the designed level [2,23,24].

Dampness is associated with mold growth on building materials, which can cause or exacerbate health conditions [25]. Several Finnish studies has shown the deleterious effect indoor moisture and mold damage in school have on students [26,27]. Remediating damaged building is associated with decreased occurrence of health symptoms among students [26].

The current work is a continuation of studies on IEQ, health and academic performance in Finnish school buildings. Previous studies include on-site investigation of ventilation systems which shows the need for maintenance or replacement due to insufficient classroom ventilation [24]. Further work showed association between mathematics achievement and both elevated classroom temperatures and absenteeism due to ill health [28]. The school level prevalence values of students’ health and wellbeing were later assessed [29]. This study reports the results from both student and principal questionnaires to study associations between school building characteristics, IEQ, and health.

## 2. Experimental Section

### 2.1. Materials and Methods

**Figure 1:**
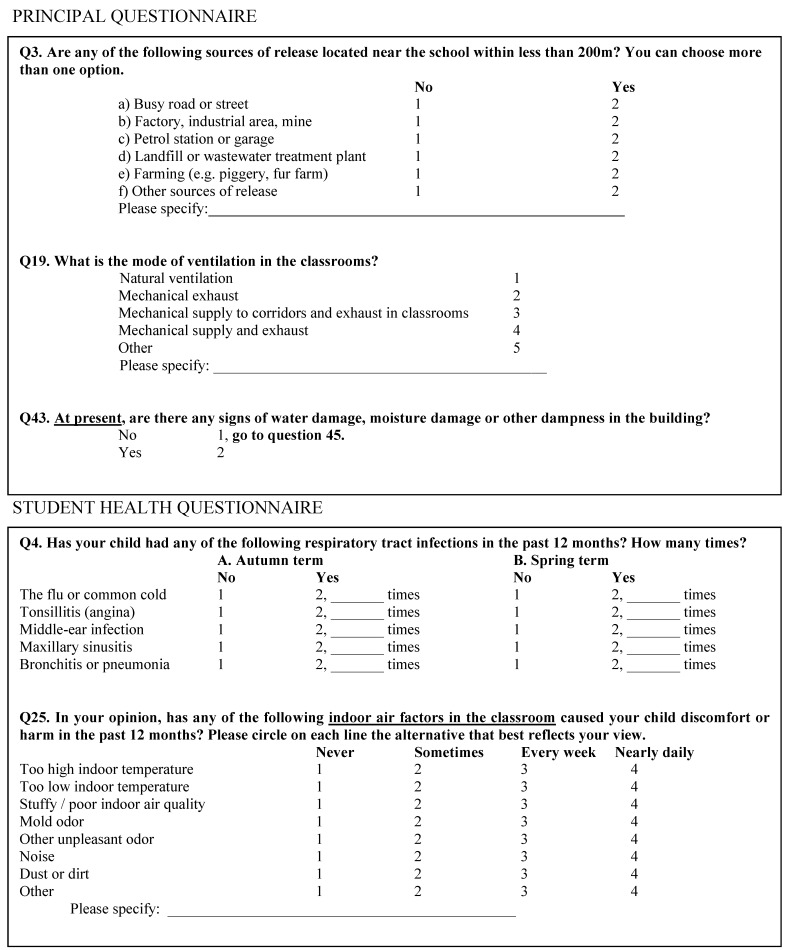
Example of questions from the principal and student questionnaires.

The data used in this study are from school principal questionnaires and student health questionnaires. A total of 2769 validated questionnaires [28] were administered in December 2007 to all Finnish elementary school principals, and 1154 of them were returned (response rate of 42%). Fifty one IEQ related questions were asked, including questions on classroom ventilation, temperature, and observations of moisture damage. Also, some basic information was asked about personnel working in the schools. The principals were encouraged to ask for help from their school technical personnel or the municipality when answering the questions. Few examples of the questions asked are shown in Figure 1.

A total of 6787 sixth grade student from 334 randomly (stratified) sampled schools received a validated questionnaire [28] comprising 37 questions relating to their home environment, school environment, social economic status, health, and wellbeing in May 2007. The questionnaires were answered by the students with the help of their parents. The response rate was estimated at 63% (4248 students from 297 schools) [29]. Examples of some of the questions asked are also shown in Figure 1.

The study was conducted in accordance with the Declaration of Helsinki, and the protocol was approved by the “Institutional review board of the National Public Health Institute, Finland, decision 4/2007 § 51 and study plan number 378”. Approval was given on the 24 April 2007. Taking part in the study was made optional and research subjects were referred to anonymously throughout the study. Participants were told that responding to the questionnaire will be interpreted to be a written consent.

### 2.2. Data Analysis

The data were analyzed using IBM SPSS statistics version 21 (International Business Machines Corporation, New York City, NY, USA) and SAS version 9.3 (SAS Institute Inc., Cary, NC, USA). The descriptive statistics for continuous variables were calculated, including mean, median, minimum, maximum, and standard deviation. Correlations between variables that were not normally distributed were studied using a non-parametric method (Spearman’s rho). While the questionnaire included separate entries for respiratory symptoms and infections in the fall and spring semesters, it was noticed that student responses concerning each semester were highly correlated. Since the questionnaire responses were collected at the end of the spring semester, it was considered that the recall concerning these health outcomes in the spring semester was better, and data concerning fall semester did not appear to bring additional value. Therefore, the analyses focused on respiratory symptoms and infections reported in the spring semester.

The association between selected IEQ variables and existence of upper respiratory symptoms (stuffy nose, rhinitis, dry or sore throat, hoarseness, dry cough, cough with phlegm, or fever over 37 °C), general symptoms (headache, fatigue, or difficulties in concentration), and missed school days due to respiratory infections among the school children was first modeled using generalized linear mixed modelling (GLMM). Existence of lower respiratory symptoms (wheezing, cough with wheezing, dyspnea) were not modelled due to low prevalence of symptoms observed.

The school intercept term was used to account for the dependence among the children at the same school. The model with the random effect (school), and dampness or mold at home, as well as exposure to environmental tobacco smoke (ETS) at home as children’s background variables, was used as the zero-model. Then, different IEQ variables, including ventilation adequacy, thermal conditions, and dampness or mold, were added to the model one by one. The Akaike Information Criterion (AIC) was used to determine which variable was the most suitable for the current model [30]. After the most suitable variable was chosen, the same procedure was used for the rest of the variables. This procedure was continued until there were no more statistically significant variables to add to the model. The *p*-value of 0.05 was used as the threshold value. The adequacy of the model was assessed by checking how well the model predicted the actual health status of the children.

Since the GLMM could not adequately predict the number of reported symptoms and missed school days due to respiratory infections, the following modelling was conducted using the hierarchical Zero Inflated Poisson (ZIP) models. ZIP models can be used when there are more zeroes in the model than expected. In the ZIP model there are two processes; the first process generates the zeroes with certain probability p and the second process can generate any possible number with the probability of 1-p.The model was built in the same way than in the case of the existence of the health symptoms. The adequacy of the model was checked by calculating the expected frequencies of the different number of symptoms and comparing them to the actual frequencies in the data.

## 3. Results

The principals who responded to the questionnaire had stayed for an average of 12.6 years in their respective schools, serving an average of 10 years as a principal (Table 1).

The number of buildings in each school studied was either one or two, with a single or multiple classrooms. The year of construction has a mean, median and range of 1981, 1988 and 125 years respectively. Most of the schools had two floors (38%), while 32%, 24% and 6% of the schools have one, three, or more than three floors respectively.

Table 2 shows the location of the schools and the facilities available in the school building. More than half of the schools (58%) were located within 200 m to a busy road, while those located within 200 m to a factory or mine, waste treatment plant, and farm were less than 20%. Only 392 (34%) of the schools had no basement, while 125 (11%) had classrooms in the basement. Others used the basement for activities such as sport, dining, and storage facilities.

Hot-water radiator was the major heat distribution method employed by the schools sampled (91%). Some 77% of the schools have had their heating systems adjusted to conform to standard.

Different ventilation types were employed by the schools studied: majority of the schools (55%) used mechanical supply and exhaust ventilation, 18% had natural ventilation, 17% had mechanical exhaust ventilation, 4% mechanical supply to corridors and exhaust in classrooms, while the remaining 6% of the schools studied had other ventilation types such as opening and closing of windows for ventilation, the use of fan and window opening, and so on. Adjustment or balancing of ventilation was done recently in 79% of the schools with mechanical ventilation systems.

Between 60% and 62% of schools with mechanical exhaust and/or supply ventilation had their ducts cleaned. The filters in the air supply units are cleaned twice a year in some schools (32%), once a year in 43% of the schools, and less often in 25% of the schools sampled. Ten percent of the schools had classroom-specific adjustable air supply units and 32% of the schools studied had trickle vents on the exterior walls or on the classroom windows. Some 51% of the principals stated that their school classrooms are aired during recess (typically 15 min break after each 45 min class), 32% ventilate classrooms occasionally, while 17% were not aired during break time.

Reported thermal conditions during heating and non-heating periods are also shown in Table 2. During heating periods, majority of school principals (89%) reported classroom temperature comfortable, and 82% of them also agreed with the temperature outside the heating period.

Ventilation was reported by the principals to be adequate in 62% of the schools. It was noticed that ventilation adequacy was significantly correlated with thermal conditions during heating season (chi square test *p*-value 0.005); classroom temperatures during heating season were reported satisfactory in 69% of schools with adequate ventilation.

Dampness or moisture damage was reported in 27% and visible mold in 4% of school buildings during the school year corresponding to the health questionnaire (2006–2007). Dampness or moisture damage correlated significantly with visible mold observations (*p* < 0.001, *r* = 0.255): only a few schools with dampness or moisture damage did not report visible mold. Some 17% of the schools reported mold odor while 36% of them had previously had mold on their surfaces. Among the buildings with visible mold, 83% had a basement, and 78% of those with mold odor also had a basement.

We also checked associations between thermal conditions, ventilation adequacy and dampness or mold, as reported by principals, and students’ perceived discomfort related to the classroom conditions, *i.e*., too hot or cold, stuffiness/poor indoor air quality (IAQ), and mold odor. There was a significant association between thermal conditions during heating season and students’ perceived high classroom temperature (OR: 1.25, 95%CI: 1.01–1.56). Statistically significant associations were also observed between inadequate ventilation and both students’ perceived stuffiness/poor IAQ (OR: 1.76, 95%CI: 1.48–2.09) and high classroom temperatures (OR: 1.73, 95%CI: 1.45–2.05). In addition, there were significant associations between students’ perceived mold odor and principals’ reported dampness or mold (OR: 3.49, 95%CI: 2.36–5.15), visible mold (OR: 3.87, 95%CI: 2.23–6.71), and mold odor (OR: 2.43, 95%CI: 1.59–3.69).

**Figure 2:**
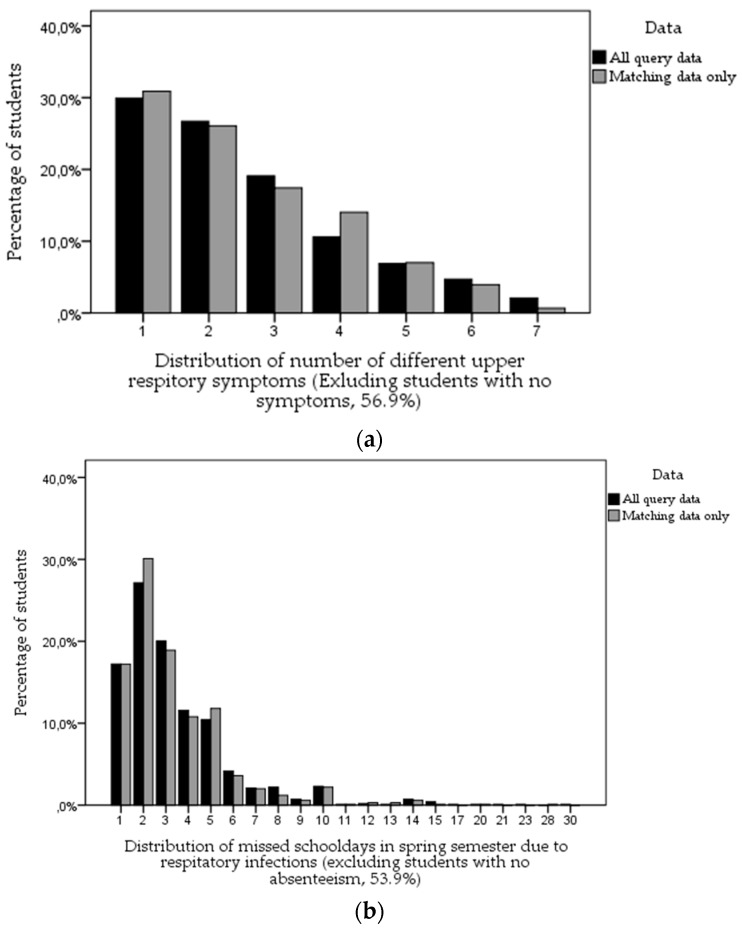
(**a**) Distribution of number of upper respiratory symptoms; (**b**) Distribution of missed school days due to respiratory infections.

Table 1 and Figure 2a,b show the distributions of number of upper respiratory symptoms and missed school days due to respiratory infections in the spring semester. There were no significant differences between all query data and sample of schools with matched principal questionnaire data. The majority of the students did not report any upper respiratory symptoms (57%) and were present throughout the period (54%). Some 11% of students reported at least two different types of upper respiratory symptoms, and 5% reported four symptoms or more. Among those that missed school due to health symptoms, there was an average of 3.4 missed school days with 29% missing two school days.

Table 3 shows fitted models predicting existence and number of upper respiratory symptoms and missed school days due to respiratory infections. Using GLMM, unsatisfactory temperature during heating season, dampness or moisture damage, and visible mold were associated with existence of upper respiratory symptoms. These variables explained 70% of the variation observed between schools.

Based on the following analysis utilizing hierarchical ZIP-models, the number of upper respiratory symptoms was significantly higher in schools where ventilation was reported inadequate or had reported dampness or moisture damage during the school year. These factors explained 44% of the variation observed between schools in the first process (zeroes), while they explained 9.5% of the variation observed between schools in the second process (counts).

School level variables were not significantly associated with missed school days due to respiratory infections in the GLMM model. However, in the ZIP-model, the number of missed school days associated significantly with ventilation adequacy: RR for higher number of missed school days due to respiratory infections due to inadequate ventilation was 1.16 (95%CI: 1.03–1.31) as compared to adequate ventilation. In the second process this factor explained about 8% of the variation observed between schools. Finally, we modelled associations between school level variables and general symptoms (headache, fatigue, or difficulties in concentration), but there were no associations by either GLMM or ZIP models (data not shown).

## 4. Discussion

From the results, the principals can be said to have enough knowledge about their respective school buildings considering the number of years they have been working there. Nevertheless, their responses may be subjective. Principals may not be considered indoor environment specialists, hence they were encouraged to ask for support from technical personnel as needed. The response rate to the principal questionnaire was moderate at 42%. However, the number of respondents (1154) and the fact that they represent the entire Finnish elementary school building stock, provides a robust dataset for our analysis.

The majority of the schools are situated 200 m or more from a source of air pollutant such as factories, petrol stations, waste treatment plants, and farmlands. However, a number of them are close to busy roads which can be a source of particulate matter and noise [3,20]; this could cause or exacerbate a health problem in students [31,32] and also affect their academic performance [33]. Also, emissions from industries and allergens from farm produce could affect air quality and be a source of odor. Further investigation is needed to show if schools closer to busy roads and industries reported more noise and poor IAQ.

Majority of the school principals reported adequate thermal comfort in classroom during both heating and non-heating periods. This could be related to general building operation in Finnish schools, as well as the recent adjustments done for the heating system to conform to standard. Also, the use of hot-water radiator for heating distribution by most of the schools (91%) encourages temperature adjustment to requirement at any particular time. The radiators can be controlled locally or centrally depending on indoor temperature. Nevertheless, too cold or unstable (both hot and cold) classroom temperatures was associated with existence of upper respiratory symptoms reported by the students.

Whereas elevated classroom temperatures have been associated with student performance [28], reduced perception of air quality, and sick building syndrome (SBS) [34,35], based on our results it is also possible that too low temperatures associate with health symptoms. However, simultaneous exposure to NO_2_ and other air pollutants (which were not analyzed in this study) may confound the association. A previous study found low temperature to influence the emergence of some respiratory disease in day care centers [36]. Other studies have found cold temperatures to affect respiratory health [37,38].

Earlier studies had indicated that the use of mechanical supply and exhaust ventilation system type is associated with a higher ventilation rate per student and ventilation per m^2^ when compared to other ventilation system types [18,19]; just about half of the principals who responded to the questionnaire confirmed having this type of ventilation system in their school.

Inadequate ventilation was associated with upper respiratory symptoms. Sufficient classroom ventilation ought to reduce exposure to bioaerosols such as mold spores and bacteria, thereby lowering the rate of respiratory illnesses. A relationship between upper respiratory symptoms and a low ventilation rate has been reported by Mi *et al.* [20].

Absenteeism has been shown to occur in school due to respiratory problems [7] and it has also been related to ventilation inadequacy [23]. In this study, there was a significant relationship between inadequate ventilation in classrooms and number of missed school days due to respiratory infections, similar to results documented by Simons *et al*. [39].

A significant correlation existed between dampness or moisture damage and visible mold. While majority of schools with visible mold observations also had reported dampness or moisture damage, it indicates that reporting of visible mold observations is a valid indicator of IEQ problems related to dampness or moisture damage. However, a large proportion of schools with reported dampness or moisture damage did not have visible mold, which should be considered in future large scale surveys, where questionnaires are often considered cost-efficient and less labor intensive than on-site building investigations. The majority of the schools with either visible mold or mold odor had basement in their building supporting recent studies that the basement of buildings is one of the usual places for indoor mold growth [40]; proper maintenance and moisture control in these high risk areas should be emphasized.

The association between dampness or moisture damage and visible mold with upper respiratory symptom before (OR: 1.33 95%CI: 1.04–2.00) or at the time of the study (OR: 1.80 95%CI: 1.13–2.87) agrees with a recent study by Borràs-Santos and colleagues [41] that suggested a higher risk of nasal symptoms (OR: 1.34, 95%CI: 1.05–1.71) for Finnish student exposed to dampness or mold in school. But they also reported association between dampness or mold and lower respiratory symptoms (OR: 1.36 95%CI: 1.04–1.78 for wheeze), which could not be confirmed due to low prevalence of symptoms observed in this study. It should be noted that our sample of schools was based on a stratified random sample, whereas the sample used by Borràs-Santos *et al.* included equal numbers of study schools and controls schools (*i.e*., schools with dampness, moisture damage or mold oversampled).

There were statistically significant associations between too hot indoor temperatures and ventilation inadequacy as reported by the principals and too hot classroom temperatures and stuffiness/poor IAQ perceived by the students, as well as between dampness or moisture damage, visible mold, and mold odor reported by the principals and mold odor perceived by the students. These associations would generally support the findings of this study regarding the reported IEQ issues.

The results are based on questionnaire responses from both principals and students, which is the main limitation of this study. It should be noted that the students responded to their questionnaires independently from the school principals in the spring of 2007, and the school principals responded to the principal questionnaires at the end of 2007. Moreover, the school principals were not aware of the results of the health questionnaires. Therefore, reporting bias should not play a major role in the observed associations between IEQ and health outcomes. Other limitations include possible influences of other factors, such as the effect of number of individuals in a classroom on respiratory symptoms, which should be considered in further studies.

## 5. Conclusions

Unsatisfactory classroom temperature during heating season, inadequate ventilation, and dampness or moisture damage, and/or visible mold, all reported by school principals, were associated with both existence and number of upper respiratory symptoms reported by sixth grade students. In addition, inadequate ventilation associated with number of missed school days due to respiratory infections. The previously described IEQ indicator variables could explain a large part of the school level variation observed in the self-reported upper respiratory symptoms and missed school days due to respiratory infections among students.

## Figures and Tables

**Table 1:** Background information of sampled schools and selected health outcomes of students (including all students responding to the questionnaire, *N* = 4248).

Attribute	Mean	Median	SD	Min	Max
Years as principal	10.0	8	7.6	1.0	36.0
Years in the building	12.6	11.0	8.6	0.5	37.0
Number of teachers in school	11.0	7.0	10.8	1	100
Number of other staffs	7.0	5.0	8.8	0	120
Number of pupils	156.9	97	274.5	9	8011
Buildings per school	1.4	1.0	0.5	1	2
School surface area (m^2^)	2343.1	1288.0	4243.7	77	74,764
Number of classrooms	10.8	7.0	9.4	1	61
Number of floors	2.1	2.0	.9	1	4
Year of construction	1981	1988	21.2	1880	2005
Number of upper respiratory symptoms in spring semester (*including students with no absenteeism*)	1.1	0.0	1.6	0	7
Number of upper respiratory symptoms in spring semester *(excluding students with no absenteeism)*	2.6	2.0	1.5	1	7
Missed school days due to respiratory infections in spring semester *(including students with no absenteeism)*	1.6	0.0	2.5	0	30
Missed school days due to respiratory infections in spring semester *(excluding students with no absenteeism)*	3.4	3.0	2.6	1	30

**Table 2:** Characteristics of the schools.

Attribute	Number of Responses	% Yes
Close proximity (<200 m) to		
Busy roads	1140	58
Factory/mine	1108	7
Petrol station/garage	1100	12
Landfill/waste treatment plant	1101	1
Farming (piggery, fur farm *etc*.)	1104	15
School facilities		
Kitchen	1147	87
Sports hall	1136	75
Assembly hall	1037	43
Language studio	1033	3
Arts and craft room	1137	83
IEQ indicator variables		
During heating season		
Comfortable	1042	89
Too cold	592	35
Too hot	539	20
Draught	635	44
Outside of heating season		
Cold floor surfaces, corners *etc*.	643	52
Comfortable	932	82
Too cold	496	12
Too hot	683	57
Draught	522	22
Cold floor surfaces, corners *etc*.	522	21
Dampness or moisture damage	1129	27
Mold present on building surfaces or structures	1130	4
Perceived mold odor in building (presently)	1124	17
Mold previously present on building surfaces	1154	
2007 summer or later		1
2006–2007 school year		3
In the past 5 years		13
Not in the specified period		19
Adequate ventilation	1138	62

**Table 3:** Fitted models predicting (1) upper respiratory symptoms and (2) missed school days due to respiratory infections. Variables not selected (NS) did not significantly improve the model.

Attribute	GLMM-Model	ZIP-Model	
		Zeroes	Counts
1A. Reported upper respiratory symptoms		1B. Number of symptoms	
Independent variables	OR (95%CI)	OR (95%CI)	RR (95%CI)
Classroom temperature during heating season		NS	NS
Satisfactory	1		
Too hot	2.21 (0.78–6.21)		
Too hot and cold	1.45 (1.04–2.00)		
Too cold	1.27 (1.03–1.58)		
Adequate ventilation	NS		
Yes		1	1
No		0.78 (0.62–0.99)	1.09 (0.96–1.23)
Dampness or moisture damage			
Never	1	1	1
Before 2006–2007	1.33 (1.00–1.77)	0.80 (0.58–1.09)	1.03 (0.86–1.22)
During 2006–2007	1.80 (1.13–2.87)	0.60 (0.38–0.95)	1.07 (0.84–1.35)
After 2006–2007	0.92 (0.56–1.50)	1.01 (0.59–1.74)	0.87 (0.64–1.17)
Visible mold		NS	NS
Never	1		
Before 2006–2007	0.77 (0.62–0.97)		
During 2006–2007	0.63 (0.35–1.13)		
After 2006–2007	2.35 (1.03–5.32)		
Dampness or mold at home	1.14 (0.83–1.55)	0.92 (0.65–1.31)	1.12 (0.96–1.31)
ETS at home	2.47 (1.58–3.85)	0.34 (0.19–0.62)	1.10 (0.91–1.35)
2A. Missed school days due to respiratory infections		2B. Number of days missed	
Adequate ventilation	NS		
Yes		1	1
No		0.97 (0.78–1.21)	1.16 (1.03–1.31)
Dampness or mold at home	1.16 (0.86–1.58)	0.90 (0.65–1.25)	1.20 (1.06–1.35)
ETS at home	0.84 (0.56–1.28)	1.24 (0.80–1.92)	1.50 (1.28–1.76)
